# Intersetorialidade na Política Paulista de Prevenção das Mortes Violentas de Crianças e Adolescentes: uma análise da *Lei nº 17.652/2023*

**DOI:** 10.1590/0102-311XPT220523

**Published:** 2024-11-11

**Authors:** Fernanda Lopes Regina, Maria Fernanda Tourinho Peres

**Affiliations:** 1 Faculdade de Medicina, Universidade de São Paulo, São Paulo, Brasil.

**Keywords:** Colaboração Intersetorial, Violência, Estratégias de Enfrentamento, Política Pública, Intersectoral Collaboration, Violence, Coping Strategies, Public Policy, Colaboración Intersectorial, Violencia, Estrategias de Enfrentamiento, Política Pública

## Abstract

O objetivo deste trabalho é identificar e analisar os obstáculos à intersetorialidade na Política Paulista de Prevenção das Mortes Violentas de Crianças e Adolescentes no Estado de São Paulo, Brasil, instituída pela *Lei nº 17.652/2023*, no âmbito de ações do Comitê Paulista pela Prevenção de Homicídios na Adolescência. Trata-se de uma pesquisa qualitativa com emprego da técnica do estudo de caso, utilizando como instrumentos para coleta de dados: entrevistas, observação participante, diário de campo e análise de documentos. Os resultados demonstraram a existência de entraves para a efetivação da intersetorialidade na referida Lei, diante da setorização presente tanto no *Projeto de Lei nº 382/2022*, que a originou, quanto diante dos vetos em sua versão final, promulgada pelo governo em março de 2023. Políticas intersetoriais devem possuir instrumentos para garantir a articulação dos setores, definindo claramente as responsabilidades dos envolvidos. Além disso, é fundamental reconhecer os limites das competências atribuídas ao Legislativo na proposição de leis.

## Abstract (en)

This study aims to identify and analyze the obstacles to intersectorality in the São Paulo Policy for the Prevention of Violent Deaths of Children and Adolescents in the State of São Paulo, Brazil, established by *Law n. 17,652/2023*, within the scope of the actions of the São Paulo Committee for the Prevention of Homicide in Adolescence. This is a qualitative study using the case study technique based on: interviews, participant observation, field diary and document analysis. The results showed that obstacles to the implementation of intersectorality in this Law exist, given the sectorization present both in *Bill n. 382/2022*, which originated it, and the vetoes in its final version, enacted by the government in March 2023. Intersectoral policies must have instruments to guarantee the articulation of sectors, clearly defining the responsibilities of those involved. In addition, it is of fundamental importance to recognize the limits of the powers attributed to the legislature when proposing laws that aim to establish them.

## Abstract (es)

El objetivo de este artículo fue identificar y analizar los obstáculos a la intersectorialidad en la Política Paulista de Prevención de Muertes Violentas de Niños, Niñas y Adolescentes en el estado de São Paulo, Brasil, que dispone la *Ley nº 17.652/2023* en el marco de las acciones del Comité de São Paulo para la Prevención de Homicidios en la Adolescencia. Se trató de una investigación cualitativa mediante un estudio de caso, en la cual utilizó entrevistas, observación participante, diario de campo y análisis documental para la recolección de datos. Los resultados mostraron que existen obstáculos a la efectividad de la intersectorialidad en la Ley ante la sectorización presente tanto en el Proyecto de *Ley nº 382/2022* que la originó como en los vetos a su versión final promulgada por el gobierno en marzo de 2023. Las políticas intersectoriales deben contar con instrumentos que aseguren la articulación de los sectores, definiendo claramente las responsabilidades de los involucrados. Además, es imprescindible reconocer los límites de las facultades atribuidas al Legislativo en la institución de leyes.

## Introdução

Há décadas, os homicídios de jovens têm permeado as discussões de estudiosos, da sociedade civil e de governantes no Brasil ^1^ ^,^ ^2^ ^,^ ^3^. No caso específico do Estado de São Paulo, embora tenha ocorrido uma queda nas taxas de homicídios, é justamente entre essa população, sobretudo negra e do sexo masculino, que elas permanecem mais elevadas ^4^.

Entre 2015 e 2021, foram registradas 24.431 mortes decorrentes de homicídio, latrocínio e lesão corporal seguida de morte no Estado de São Paulo. Desse total, 2.122 vítimas eram crianças e adolescentes com idades entre 10 e 19 anos. Em 2021, jovens nas faixas etárias de 15 a 19 e de 20 a 29 anos estiveram entre as principais vítimas, com taxas que chegaram a 5,4 e 10,8 por 100 mil habitantes, respectivamente. Jovens do sexo masculino foram mais vitimizadas por intervenção policial, correspondendo a 100% dos casos no mesmo ano, sendo que 63,4% deles eram negros ^5^.

Nesse sentido, as características da administração pública e seu impacto nas políticas influenciam nas respostas ao problema, sobretudo no que diz respeito ao desafio de constituir políticas públicas intersetoriais ante a dificuldade de integração entre: os níveis de governo e os setores; a fragmentação e a descontinuidade das políticas; e os problemas decorrentes dos processos decisórios, a implementação e a avaliação das políticas ^6^ ^,^ ^7^ ^,^ ^8^.

Desde que a Organização Mundial da Saúde (OMS) declarou a violência como um dos principais problemas de saúde no mundo, diversas iniciativas buscam abordar a temática a partir da perspectiva enunciada, de que a violência pode e deve ser prevenida, sendo essa uma tarefa de toda a sociedade ^9^.

Para reduzir a vitimização letal de jovens, a OMS reforça continuamente seu papel junto a governos locais, concentrando esforços em ações que tenham como objetivo a prevenção dos homicídios. Diferentes publicações demonstram as dificuldades para identificar como a juventude e os contextos sociais relacionam-se e aumentam ou diminuem as chances de exposição à violência ^9^ ^,^ ^10^ ^,^ ^11^.

A violência é caracterizada como um fenômeno inserido em uma rede composta por fatores sociais, culturais, políticos, entre outros, que concorrem para sua natureza complexa e polissêmica ^1^. Nesse sentido, diferentes autores do campo da saúde argumentam que o trabalho intersetorial é fundamental para sua prevenção ^3^ ^,^ ^12^.

O conceito de intersetorialidade foi introduzido no campo da saúde a partir da Conferência Internacional sobre Cuidados Primários de Saúde, ocorrida em 1978 em Alma-Ata (atual Almati, Cazaquistão), em que se declarou a necessidade de estratégias suficientemente abrangentes que não envolvessem apenas a prestação de serviços em si, mas que também abordassem as questões sociais, econômicas e políticas que interferem nos agravos de saúde ^13^.

No Brasil, a intersetorialidade ganhou força a partir da promulgação da *Constituição Federal* de 1988, por meio da inauguração do conceito de seguridade social, baseada no tripé Saúde-Previdência-Assistência Social. Nesse contexto, surgiu o Sistema Único de Saúde (SUS), no mesmo ano de promulgação da Constituição, e, posteriormente, o Sistema Único de Assistência Social (SUAS), em 2005 ^14^.

Assim, uma das maiores contribuições do SUS à intersetorialidade veio por meio da conformação e da operacionalização das Redes de Saúde, constituídas por serviços que têm objetivos definidos de forma coletiva entre as instituições e que se articulam para alcançar as soluções de problemas, reforçando um dos princípios centrais do sistema: a integralidade ^15^ ^,^ ^16^.

Seguindo as orientações da OMS, o Ministério da Saúde declarou a violência como um grave problema para a saúde pública e, assim, criou políticas para o seu enfrentamento. Entre elas, há a Política Nacional de Redução da Morbimortalidade por Acidentes e Violências (*Portaria nº 737/2001*), criada em 2001, mesmo ano em que se definiu um instrumento de notificação no caso de suspeita ou confirmação de violências contra crianças e adolescentes (*Portaria nº 1.968/2001*). Em 2003, foi constituída a Política Nacional de Atenção às Urgências (*Portaria nº 1.863/2003*) ^15^.

Em 2010, como desdobramentos dessas iniciativas, o Ministério da Saúde lançou o documento *Linha de Cuidado para a Atenção Integral à Saúde de Crianças, Adolescentes e suas Famílias em Situação de Violências: Orientação para Gestores e Profissionais de Saúde* ^15^, caracterizado como uma estratégia para impulsionar o cuidado, a proteção e a defesa dessas populações por meio de redes setoriais de serviços de saúde e da rede intersetorial.

O emprego da intersetorialidade baseia-se no fato de que, com maior frequência, observa-se a ineficiência da implementação de políticas setoriais dada a crescente complexidade dos problemas sociais emergentes e da insuficiência de recursos disponibilizados para tal. Assim, a intersetorialidade tornou-se uma estratégia utilizada frequentemente para atuar contra as iniquidades sociais, além de mostrar-se mais efetiva e menos onerosa e também por possibilitar a articulação de diferentes setores da administração pública e deles com a sociedade civil ^17^ ^,^ ^18^ ^,^ ^19^ ^,^ ^20^.

Entretanto, o conceito de intersetorialidade e sua aplicação esbarra em desafios teóricos e, sobretudo, práticos. Em relação ao primeiro, ao realizarem a revisão da literatura científica sobre o tema, com estudos publicados a partir de 1994, Dubois et al. ^21^ indicaram que existem diferentes definições do conceito, de modo que não é possível verificar homogeneidade entre elas. Apesar disso, a definição adotada pelo trabalho é aquela proposta por Junqueira & Inojosa ^22^ (p. 27), para os quais ela é uma “*articulação de saberes e experiências no planejamento, realização e avaliação de ações para alcançar efeito sinérgico em situações complexas, visando o desenvolvimento social, superando a exclusão social*”.

Em relação aos desafios práticos, a literatura reconhece que a intersetorialidade não surge de maneira espontânea nas instituições, pois as diferentes características dos atores, como formações, identidades profissionais, trajetórias, valores, entre outros, deixa implícito que as ações intersetoriais envolvem certo grau de conflito ^23^.

Assim, com o intuito de discutir essas questões, este artigo objetiva identificar e analisar os obstáculos à intersetorialidade na Política Paulista de Prevenção das Mortes Violentas de Crianças e Adolescentes por meio da análise da *Lei nº 17.652*, de 17 de março de 2023 ^24^, e seus vetos.

## Metodologia

Este artigo é um recorte da tese de doutorado intitulada *Intersetorialidade para a Prevenção dos Homicídios: Um Estudo da Construção do Comitê Paulista pela Prevenção de Homicídios na Adolescência*. Trata-se de uma pesquisa qualitativa que empregou a técnica do estudo de caso para analisar o Comitê Paulista pela Prevenção de Homicídios na Adolescência (CPPHA), compreendendo quatro fontes de dados: (i) entrevistas; (ii) observação participante; (iii) diário de campo; e (iv) análise de documentos.

Para as entrevistas (20 no total), foram selecionados um representante de cada setor que participava do CPPHA, como representantes de secretarias de governo e organizações não governamentais (ONG) que atuam na temática de violência e juventude, buscando abarcar as instituições do governo e a sociedade civil, e foram aplicados questionários semiestruturados. As entrevistas continham questões sobre: características dos entrevistados (sexo, função, tempo na instituição, cargo); como as diferentes instituições estão ou deveriam estar inseridas em ações para prevenir homicídios; quais ações desempenham (ou deveriam desempenhar) para tal; e como identificam e buscam superar os limites do trabalho intersetorial para a prevenção. Diante do quadro da pandemia da COVID-19, elas foram realizadas em meio virtual pela plataforma Google Meet (https://meet.google.com). Todas foram gravadas, iniciando após consentimento expresso pelos entrevistados, para os quais foi enviado um Termo de Consentimento Livre e Esclarecido (TCLE) para assinatura.

Entre fevereiro de 2021 e dezembro de 2022, foram feitas observações das reuniões do Conselho Participativo, espaço em que se colocavam as questões a serem debatidas e a proposição de projetos e programas do comitê, por meio da discussão entre profissionais das secretarias e instituições de justiça; sociedade civil e demais interessados. O conselho era constituído por quatro grupos de trabalho (GT).

O GT1 tinha como objetivo o levantamento e a análise de dados para subsidiar os trabalhos dos demais GTs. Em sua composição, predominaram membros da academia e de institutos de pesquisa, com participações pontuais de representantes de outros setores. Ao GT2, atribuía-se o papel de desenvolvimento e articulação de políticas públicas para a redução e a prevenção de mortes de adolescentes. Esse GT contou, em sua composição, com diferentes atores governamentais e da sociedade civil.

O GT3 teve como objetivo mapear, identificar e fortalecer os atores e coletivos que atuam nos territórios mais vulneráveis a homicídios de jovens, buscando reunir a sociedade civil e o poder público para sua prevenção. O grupo contou, em sua maioria, com representantes da sociedade civil. O GT4 era responsável pela construção de protocolos, normativas e programas para a efetivação dos direitos de adolescentes, sendo majoritariamente constituído por instituições de justiça, segurança pública e, em menor número, organizações da sociedade civil.

Cabe ressaltar que a pesquisadora era integrante dos GTs, na condição de participante - representante da Faculdade de Medicina da Universidade de São Paulo -, desde o início das atividades do CPPHA. Para o período de outubro de 2019 a fevereiro de 2021 - não compreendido pela observação participante -, foram utilizadas na análise os relatórios das reuniões.

As reuniões dos GTs aconteceram mensalmente em meio virtual, nas plataformas Microsoft Teams (https://teams.microsoft.com) e Google Meet. Foi concedido à pesquisadora breve tempo no início de uma reunião em cada GT para que fosse feita a apresentação da pesquisa e os participantes fossem informados sobre a observação e pudessem consentir. Com o acordo de todos, o e-mail dos presentes foi informado pelo chat, para o envio dos TCLE a serem assinados.

O diário de campo foi empregado como um instrumento de coleta de dados em que foram anotadas sistematicamente as percepções, as descrições de atividades, os acontecimentos, as conversas e os questionamentos nas observações realizadas. A partir dele, buscou-se identificar: setores participantes e os seus respectivos envolvimentos com a agenda; posicionamentos institucionais; temas emergentes e como eles eram discutidos; as propostas de ações e estratégias surgidas para o enfrentamento dos homicídios e suas evoluções.

Por fim, foram consultados documentos para obter outros tipos de informações e evidências sobre: o entendimento sobre o problema; o estabelecimento formal das competências e das práticas dos setores envolvidos; a obtenção de dados oriundos de estudos realizados pelo CPPHA ou pelas instituições/organizações integrantes; as informações de documentos administrativos e das atas das reuniões dos GTs, entre outras.

Neste artigo, o foco é a análise da *Lei nº 17.652/2023*, que institui a Política Paulista de Prevenção das Mortes Violentas de Crianças e Adolescentes e seus vetos. Dessa forma, os dados produzidos por meio das entrevistas, da observação e do diário de campo foram utilizados apenas com o objetivo de contextualizar o CPPHA. Além disso, ênfase foi dada à análise documental, mediante análise de conteúdo. Foram feitas leituras verticais e transversais dos documentos, identificadas categorias recorrentes e sistematizadas as informações em torno dos vetos e seus argumentos.

### Aspectos éticos

A pesquisa foi aprovada pelo Comitê de Ética em Pesquisa do Hospital das Clínicas da Faculdade de Medicina da Universidade de São Paulo (HCFMUSP; parecer nº 4.565.315).

## Resultados e discussão

### O contexto para a formulação, a proposição e a aprovação da *Lei nº 17.652/2023*

Em 10 de dezembro de 2018, com a assinatura de um Protocolo de Intenções (Parecer Jurídico: AJG 221/2018) entre a Assembleia Legislativa do Estado de São Paulo (ALESP), o Fundo das Nações Unidas para a Infância (UNICEF) e o governo do Estado de São Paulo - então representado pela Casa Civil e, posteriormente, pela Secretaria de Justiça e Cidadania -, foi formalizada a criação do CPPHA. Os Comitês Estaduais de Prevenção de Homicídios são experiências vinculadas à Plataforma dos Centros Urbanos do UNICEF, presentes em três outras Unidades da Federação: Ceará, Bahia e Rio de Janeiro.

As primeiras tratativas para sua implementação tiveram início em 2019, quando foi formalizada a composição legislativa do CPHHA e sua presidência, com deputados de cinco partidos: Rede Sustentabilidade - assumindo a presidência do comitê - Democratas (DEM), Partido Social Liberal (PSL), Partido Comunista do Brasil (PCdoB) e Mandata Ativista ^25^. No dia 5 de setembro de 2019, ocorreu a cerimônia de lançamento do CPPHA na ALESP, com a participação de autoridades governamentais, representantes da sociedade civil organizada, movimentos sociais, pesquisadores e demais pessoas interessadas e engajadas no tema.

As atividades iniciaram-se em 2020, pautadas em uma perspectiva interdisciplinar e intersetorial, conjurando esforços do Legislativo, do Executivo, do Judiciário e da sociedade civil para o enfrentamento dos homicídios de adolescentes entre 10 e 19 anos, com o objetivo de construir protocolos, indicadores, recomendações, planos e programas de prevenção ^19^. As instituições presentes estão representadas no Quadro 1.

**Quadro 1:** Instituições e participantes do Comitê Paulista pela Prevenção de Homicídios na Adolescência (CPPHA). Estado de São Paulo, Brasil.

SETOR	INSTITUIÇÕES
Secretarias e órgãos do Estado de São Paulo	Saúde; Educação; Desenvolvimento Econômico; Justiça e Cidadania; Ouvidoria da Polícia do Estado de São Paulo; Polícia Civil; Polícia Militar; Fundação Casa
Secretarias e órgãos do Município de São Paulo	Direitos Humanos; Guarda Civil Metropolitana; Conselho Tutelar; Conselho Municipal de Crianças e Adolescentes de São Paulo; Saúde
Instituições de justiça	Ministério Público do Estado de São Paulo; Defensoria Pública do Estado de São Paulo; Tribunal de Justiça do Estado de São Paulo
Universidades e grupos de estudos	Laboratório Interdisciplinar de Estudos sobre Violência e Saúde da Faculdade de Medicina da Universidade São Paulo; Universidade Federal de São Paulo; Núcleo de Estudos da Violência da Universidade de São Paulo; Grupo de Estudos sobre Violência e Administração de Conflitos da Universidade Federal de São Carlos
Institutos de pesquisa	Fórum Brasileiro de Segurança Pública; Instituto Sou da Paz; Rede de Conhecimento Social
Coletivos e organizações da sociedade civil	Rede de Resistência Contra o Genocídio; Bancada da Educação; Associação Escola Cidade Aprendiz; Mundo Aflora; Centro de Educação Popular em Direitos Humanos; Visão Mundial; Associação Nacional de Ex-Conselheiros Tutelares; Viração; Coordenadoria da Mulher em Situação de Violência Doméstica e Familiar do Poder Judiciário (Campinas); e demais pessoas sem vínculo institucional e interessadas na pauta

Fonte: elaborado a partir de Comitê Paulista pela Prevenção de Homicídios na Adolescência ^25^.

O CPPHA se organiza seguindo um modelo de governança, ou seja, de descentralização da constituição de políticas públicas em que, para além do estado, participam atores da sociedade civil. Para isso, foram estabelecidas instâncias de participação organizadas em três conselhos: (i) Executivo; (ii) Participativo; e (iii) Consultivo, além da equipe técnica.

O Conselho Participativo foi destinado ao debate e à proposição das iniciativas do CPPHA, por meio da discussão entre profissionais de secretarias e instituições de justiça, sociedade civil e demais participantes (Quadro 1). Ele foi organizado em quatro GTs que se reuniam mensalmente: (i) dados, métodos e pesquisa; (ii) políticas públicas interinstitucionais; (iii) território em pauta; e (iv) a justiça e o adolescente.

Durante o período compreendido pela pesquisa, o CPPHA constituiu quatro principais iniciativas. Duas delas, a consulta *Violências no Cotidiano de Adolescentes* e o gibi *Voltei pro Mundão, e Agora?*, foram protagonizadas pela sociedade civil, ao passo que a segunda envolveu, em grande medida, a Fundação Centro de Atendimento Socioeducativo ao Adolescente (Fundação Casa), além de outras, como a Secretaria de Saúde, a Secretaria de Educação e instituições de justiça.

As outras duas foram protagonizadas pelo governo: a carta-compromisso *Adolescente Seguro* e o *Projeto de Lei nº 382/2022* (PL nº 382) ^26^, que daria origem à *Lei nº 17.652/2023*, que será vista na sequência.

### O PL nº 382/2022

Inicialmente elaborado pela coordenação do CPPHA, o texto base para o PL nº 382 foi apresentado em uma reunião interinstitucional com vistas à construção da proposta de lei escrita a várias mãos, a partir da contribuição das diferentes instituições. Analisando o processo, é importante destacar que os participantes atuaram diretamente sobre a escrita do texto, levantando importantes discussões sobre os temas a serem incluídos, considerando as capacidades institucionais de suas respectivas organizações.

O PL nº 382 propunha a instituição da Política Paulista de Prevenção das Mortes Violentas de Crianças e Adolescentes, a ser coordenada, monitorada e avaliada pelo Comitê da Política Paulista de Prevenção das Mortes Violentas de Crianças e Adolescentes. Dividido em dez seções e composto de 24 artigos, o texto final do PL nº 382 apontava para a vocação intersetorial da Política Paulista de Prevenção das Mortes Violentas de Crianças e Adolescentes, quando na seção V, art. 7º, estabelece que: “*a coordenação, articulação, monitoramento e avaliação da Política Paulista de Prevenção das Mortes Violentas de Crianças e Adolescentes, prevista nesta Lei, serão executados por meio do Comitê da Política Paulista de Prevenção das mortes violentas de crianças e adolescentes, instância interinstitucional de caráter consultivo e deliberativo, que tem como finalidade assegurar a articulação das ações voltadas à prevenção à morte violenta de crianças e adolescentes, em âmbito estadual, conforme dispuser regulamento…*” ^26^.

Nos objetivos e nas diretrizes também é possível perceber a orientação intersetorial proposta, tanto horizontal quanto vertical. Na seção III, art. 5º, é estabelecido que a Política Paulista de Prevenção das Mortes Violentas de Crianças e Adolescentes tem como objetivo “*promover ações integradas e multidisciplinares para a prevenção das mortes violentas de crianças e adolescentes*” ^27^. Além disso, estão entre os objetivos “*fomentar a integração entre ações e iniciativas no âmbito estadual e municipal*” e uma atuação “*colaborativa do Estado com os municípios*”, o que aponta para o reconhecimento da importância de uma intersetorialidade vertical.

Por ser uma iniciativa estadual, os municípios apareceram timidamente tanto nos objetivos e quanto nas diretrizes, estando completamente ausentes na representação do que seria o Comitê da Política Paulista de Prevenção das Mortes Violentas de Crianças e Adolescentes, embora sejam fundamentais para a gestão de políticas públicas para as juventudes nos territórios, inclusive instituídos por meio de normativas e leis para tal função, permanecem ocupando pouco espaço na prática ^27^, o que acaba por invisibilizar os territórios.

A literatura aponta que é fundamental valorizar as populações locais no atendimento de suas demandas, pois elas constroem socialmente esses territórios e compartilham características socioeconômicas e culturais que incidem diretamente no êxito das ações intersetoriais ^28^.

As diretrizes foram apresentadas na seção IV, art. 6º, nas quais se pode notar menções diretas/explícitas ou indiretas/implícitas à intersetorialidade em quatro das 11 listadas: fomentar o planejamento e a implementação das políticas públicas de forma integrada entre as diferentes secretarias e áreas temáticas; integrar e acompanhar instituições públicas, privadas e da sociedade civil e suas ações na promoção da política de prevenção e redução da morte violenta de crianças e adolescentes; fomentar formação continuada aos profissionais de segurança pública e do sistema de justiça; e fomentar formação continuada dos profissionais da saúde, educação e assistência social e outras secretarias que atuam com crianças e adolescentes ^26^.

É importante destacar a referência à integração entre diferentes secretarias na fase de planejamento de políticas públicas. De acordo com Oliveira et al. ^29^ (p. 1310), o envolvimento de diferentes setores desde o momento do planejamento da ação é central para que se tenha uma intersetorialidade plena: “*a intersetorialidade, pressupõe uma articulação que engloba as fases de planejamento, execução das atividades e avaliação. No nível das práticas, entretanto, se observa a persistência de resistências em seu emprego, que se dão, sobretudo, em razão da dificuldade de sua efetivação diante das diferentes leituras acerca dos problemas sociais por parte dos setores, bem como pelas diferentes características dos atores envolvidos como formações, identidades profissionais, trajetórias, valores, entre outros, gerando, por sua vez, disputas de poder que podem dificultar o estabelecimento de um diálogo para a construção de ações intersetoriais*”.

Apesar disso, ainda que assumisse um caráter intersetorial, no sentido de compreender os homicídios a partir de suas diferentes causas, o PL nº 382 não previa de modo explícito o mecanismo para a articulação efetiva dos setores, ou seja, não indicava de que maneira a intersetorialidade seria operacionalizada.

Nesse sentido, mais relevante nos parece a definição de representação e responsabilidades, na seção VI, na qual são listadas as instituições que comporiam o CPPHA. O PL nº 382 instituía a composição igualitária de membros de diferentes secretarias estaduais (Justiça e Cidadania, Educação, Segurança Pública, Saúde, Desenvolvimento Social, Desenvolvimento Econômico e Esporte e Lazer), das instituições de justiça (Ministério Público do Estado de São Paulo, Defensoria Pública do Estado de São Paulo, Tribunal de Justiça do Estado de São Paulo), da ALESP, do Conselho Estadual da Criança e do Adolescente e da sociedade civil (universidades, associações, instituições que atuem nos temas previstos na Lei) ^26^. Eram também definidas as atribuições e as responsabilidades dessas instituições, conforme Quadro 2.

**Quadro 2:** Secretarias e instituições de justiça presentes no *Projeto de Lei nº 382/2022* e suas atribuições. Estado de São Paulo, Brasil.

SECRETARIAS E INSTITUIÇÕES DE JUSTIÇA	ATRIBUIÇÕES
Políticas de educação	(a) Consolidar informações sobre ocorrências disciplinares e evasão escolar por território e de forma semestral, bem como dados de frequência escolar para embasar programas de busca ativa (b) **Realizar a busca ativa de estudantes e notificar o Conselho Tutelar, em observância ao art. 56º do ECA** (c) Criar e fortalecer estratégias para monitorar a defasagem escolar (d) **Desenvolver, em parceria com a rede de saúde, programas de prevenção à gravidez na adolescência voltada a meninas e meninos** (e) Mobilizar as escolas públicas e privadas para criação das comissões de conscientização, prevenção e enfrentamento à violência e promoção dos direitos da criança e do adolescente (f) Promover programas específicos para a sensibilização da violência contra mulheres entre estudantes das instituições de ensino (g) **Promover a transição positiva do ambiente escolar para o mundo do trabalho, em articulação com as políticas de desenvolvimento econômico e trabalho, de forma que os estudantes estejam melhor preparados para o processo de inclusão socioprodutiva**
Políticas de segurança pública	(a) Divulgar trimestralmente, no formato de microdados, informações qualificadas de violência e de mortes violentas contra crianças e adolescentes, asseguradas as garantias à privacidade de informações pessoais (b) Qualificar agentes de segurança pública, principalmente os profissionais que estão no atendimento nas delegacias, em temas de direitos humanos, sociais, constitucionais, ECA, Sistema de Garantia dos Direitos da Criança e do Adolescente Vítima ou Testemunha de Violência e da *Lei nº 17.428/2021* (c) Priorizar no orçamento público o investimento na formação dos agentes de segurança pública e desenvolvimento de políticas e protocolos de segurança com enfoque na prevenção à violência e à morte violenta (d) Assegurar o imediato registro de ocorrência de crianças e adolescentes desaparecidos, priorizando a busca pelos órgãos de segurança pública, conforme preconizado no art. 208º do ECA (e) Integrar as bases de crianças e adolescentes desaparecidos com as bases de mortes violentas de crianças e adolescentes do Estado
Políticas de saúde	(a) Fortalecer e ampliar os programas de prevenção à violência, regulados pela Secretaria Estadual de Saúde, tais como o Programa de Saúde do Adolescente e o Programa de Prevenção de Agravos e Violências, além dos demais que venham a substituí-los e que tiverem relação com os objetivos desta Lei (b) Fortalecer e ampliar a estruturação da RAPS para atenção à saúde mental de crianças e adolescentes (c) Realizar a vigilância epidemiológica e o monitoramento com notificação compulsória dos casos do registro das mortes violentas de crianças e adolescentes, com análise e divulgação dos dados (d) Qualificar as informações sobre morte violenta de crianças e adolescentes, na área da saúde (e) Ampliar os atendimentos e acompanhamentos psicológicos às crianças e adolescentes que vivem em situação de tensão e risco em serviços de saúde da atenção básica (f) Atuar de forma integrada com as áreas de educação e assistência social nas estratégias de busca ativa escolar
Políticas de assistência social	(a) Atuar de forma integrada com as áreas de educação e saúde nas estratégias de busca ativa escolar (b) Inserir a capacitação sobre programas e iniciativas de políticas da prevenção e enfrentamento às mortes violentas de crianças e adolescentes e rodas de conversa nos serviços de baixa, média e alta complexidade (c) Criar programas de apoio e fortalecimento para as mães e familiares vítimas de violência contra crianças e adolescentes (d) Fortalecer os programas de prevenção e enfrentamento às mortes violentas de crianças e adolescentes (e) Implementar serviço de monitoramento pelos CREAS das causas de morte violenta de adolescentes, bem como daqueles em cumprimento de medidas socioeducativas (f) Fomentar políticas de acolhimento a crianças e adolescentes em situação de risco à morte violenta, de forma descentralizada (g) Fomentar políticas de proteção provisória e acolhida a crianças e adolescentes em situação de risco à morte violenta
Políticas de desenvolvimento econômico	(a) Garantir para todo adolescente o direito à aprendizagem, à profissionalização e ao trabalho, na forma prevista em lei (b) Desenvolver programas específicos de aprendizagem e acesso à renda para adolescentes em situação de risco social e pessoal, escolaridade defasada idade-série, dos serviços de acolhimento institucional e familiar e em cumprimento de medida socioeducativa (c) Realizar convênios com o PPCAAM para a profissionalização e acesso à renda de famílias e adolescentes acolhidos pelo programa (d) Incentivar e fortalecer as instituições da sociedade civil e empresas que trabalham com aprendizagem de adolescentes, em especial para adolescentes em cumprimento de medidas socioeducativas (e) Manter e ampliar programa de transferência de renda para as famílias, priorizando famílias que perderam no último ano um ou mais adolescentes para a morte violenta
Políticas de justiça e cidadania	(a) Fortalecer o PPCAAM em São Paulo (b) Criar unidades do CRAVI de forma descentralizadas nos territórios com maior vulnerabilidade à morte violenta de crianças e adolescentes (c) Desenvolver nos CIC, programas específicos para a prevenção à morte violenta de crianças e adolescentes (d) Instituir, no âmbito da Fundação Casa, o programa de pós medida, conferindo dotação orçamentária própria e contemplando todos os adolescentes egressos da Fundação Casa (e) Estabelecer políticas voltadas ao atendimento de casos que se enquadrem em contextos de proteção provisória
Instituições de justiça (Ministério Público, Defensoria e Tribunal de Justiça)	(a) Implementar serviço de monitoramento das causas de morte violenta de adolescentes, bem como daqueles em cumprimento de medidas socioeducativas (b) Criar banco de dados qualificados dos processos de mortes violentas contra crianças e adolescentes (c) Priorizar a tramitação de inquéritos e processos criminais relativos a mortes violentas de crianças e adolescentes e desenvolver mecanismos que viabilizem essa priorização

Fonte: São Paulo ^26^.

CIC: Centros de Integração da Cidadania; CRAVI: Centro de Referência e Apoio à Vítima; CREAS: Centros de Referência Especializado de Assistência Social; ECA: *Estatudo da Criança e do Adolescente*; PPCAAM: Programa de Proteção à Crianças e Adolescentes Ameaçados de Morte; RAPS: Rede de Atenção Psicossocial.

Embora se reconheça que o PL nº 382 buscou garantir ampla representação institucional, as atribuições foram constituídas de maneira fragmentada, “delegando” aos setores tarefas relativas às suas competências, sem que fosse estabelecida uma diretriz transversal ao que era previsto. Não obstante, para algumas ações desempenhadas por determinados setores, são estabelecidas parcerias (Quadro 2, em negrito).

Nota-se uma ênfase na articulação entre a educação e os demais setores, sendo eles saúde, assistência social, conselhos tutelares, trabalho, desenvolvimento econômico. Os dois primeiros, entretanto, são fundamentais para o desenvolvimento de ações nos territórios, aparecem juntos apenas na articulação para a busca ativa de escolares, compondo esforços com a educação.

Cabe ressaltar que, para as instituições de justiça (Defensoria, Ministério Público e Tribunal de Justiça) e segurança, não são mencionados projetos intersetoriais junto aos demais. Esse “isolamento” fica ainda mais claro quando se observam as seções VII (das políticas de segurança pública e interfaces com a proteção à vida de crianças e adolescentes) e VIII (das ações diante da ocorrência de mortes violentas de crianças e adolescentes), que apresentam atuações específicas para esses atores (Quadro 3).

**Quadro 3:** Atuações específicas para a Secretaria de Segurança Pública, Guarda Civil Metropolitana, Ministério Público, Poder Judiciário e Defensoria Pública previstas no *Projeto de Lei nº 382/2022*. Estado de São Paulo, Brasil.

SEÇÃO VII	
Polícias Civil e Militar	As instituições policiais devem expedir normativas, protocolos e ações que visem atender crianças e adolescentes, a partir de suas especificidades, com ênfase na prevenção à morte violenta deste grupo social. As operações das Polícias Civil e Militar deverão sempre atuar a partir de um plano de redução de riscos e danos para evitar violações de direitos humanos e preservar, em especial, a vida de crianças e adolescentes, observando especialmente as seguintes diretrizes: (i) uso progressivo da força e a adoção de um Procedimento Operacional Padrão específico para uma abordagem adequada e não violenta de crianças, adolescentes; e (ii) elaboração de planos de segurança pública que priorizem a proteção de crianças e adolescentes, de suas vidas, integridade física, de suas casas e espaços de educação e sociabilidade
Guarda Civil Metropolitana	As ações da Guarda Civil Metropolitana deverão observar, no que couber e no âmbito das suas competências, o disposto neste artigo
Ministério Público, Poder Judiciário e Defensoria Pública	O Ministério Público, o Poder Judiciário e a Defensoria Pública, na elaboração de suas respectivas propostas orçamentárias, poderão prever recursos para a criação e manutenção da equipe de atendimento multidisciplinar voltadas ao atendimento de crianças, adolescentes e suas famílias, relativos a episódios de mortes violentas, nos termos da Lei de Diretrizes Orçamentárias
SEÇÃO VIII	
Ministério Público	Em todos os casos de mortes violentas de crianças e adolescentes o Ministério Público deverá ser automaticamente notificado, para monitorar a prioridade e a observância à *Lei nº 17.428/2021*
Não deixa claro quem	Deve-se garantir o atendimento psicossocial gratuito às famílias que tiveram crianças e adolescentes vitimados de forma violenta
Secretaria de Segurança Pública	A Secretaria de Segurança Pública do Estado de São Paulo deve divulgar periodicamente boletins, dados e informações sobre a morte violenta de crianças e adolescentes ocorridas no estado

Fonte: São Paulo ^30^.

O PL nº 382 expressa dificuldades em articular os setores para propor ações conjuntas. Não foram construídos ou propostos caminhos para tornar a agenda de prevenção de homicídios de jovens um orientador transversal para todas as secretarias, instituições de justiça e para a sociedade civil, não apenas enquanto um tema ou problema a ser resolvido a partir das *expertises* setoriais fragmentadas, mas a partir de um instrumento mais amplo, construído a partir de um pacto colaborativo entre eles.

O principal desafio repousa no fato de que a integração entre os diferentes setores requer um esforço de entendimentos e ações coletivas sobre determinados problemas, respeitando e articulando diferentes saberes. Ainda assim, esse é um desafio que não nasce - e não se encerra - com o PL nº 382, já que a falta de integração das políticas no Brasil faz parte de uma histórica cultura organizacional.

Dessa forma, por mais que se busque enfrentar os homicídios a partir de uma perspectiva intersetorial, em grande medida, eles ainda ocupam um lugar social, institucional e normativamente focalizado, que permite compreendê-los ora pertencendo à dualidade crime-sistema criminal, ora a políticas sociais.

### Do PL nº 382 à *Lei nº 17.652/2023*: a Política Paulista de Prevenção das Mortes Violentas de Crianças e Adolescentes depois dos vetos

Em 28 de junho de 2022, foram propostas quatro emendas ao texto do PL nº 382 . Na primeira, foi solicitada a inclusão de acolhimento para crianças e adolescentes que vivem nas ruas, o que levou à inclusão da orfandade. Na segunda, a deputada autora solicitava que fosse suprimida a alínea “e” do inciso V, do art. 14º, que previa a ampliação de programas de transferência de renda para as famílias que perderam adolescentes de forma violenta. A terceira propunha a inclusão do estupro seguido de morte, como parte da categoria “morte violenta” detalhada no art. 2º. A última, por sua vez, opunha-se à criação do Comitê Paulista de Prevenção das Mortes Violentas de Crianças e Adolescentes, dado que a *Lei nº 17.347*, de 12 de março de 2021, já instituía um Comitê Estadual Intersetorial de Políticas Públicas pela Primeira Infância de São Paulo, como pode ser visto: “*esta Parlamentar reconhece a importância de haver uma equipe responsável por monitorar e coordenar a execução das políticas previstas no projeto. Ocorre que a criação de novo Comitê se mostra desnecessária. Por um lado, poder-se-ia destacar funcionários dos próprios quadros já existentes da Administração Pública para desempenhar as funções estabelecidas na propositura, haja vista que o Poder Público conta com inúmeros servidores capacitados para atuar na área. Inclusive, no âmbito da Secretaria Municipal de Direitos Humanos e Cidadania, já existe o Conselho Municipal de Direitos da Criança e do Adolescente, com composição paritária entre diversas Secretarias e entidades da sociedade civil e com atuação em políticas voltadas a esse público. Por outro lado, imperioso relembrar que a própria autora da proposta, por meio do Projeto de Lei nº 1.027/2019, convertido na Lei nº 17.347/2021, já criou um Comitê Intersetorial destinado às políticas envolvendo crianças. Leia-se: Artigo 16 - A coordenação, articulação, monitoramento e avaliação da Política Estadual pela Primeira Infância de São Paulo, previstos nesta Lei, serão executados por meio do Comitê Estadual Intersetorial de Políticas Públicas pela Primeira Infância de São Paulo, que tem como finalidade assegurar a articulação das ações voltadas à proteção e à promoção dos direitos da criança na primeira infância, em âmbito estadual, conforme dispuser regulamento. Tal Comitê, inclusive, tem composição muito similar ao que se pretende criar, com representantes da Casa Civil, Secretaria de Educação, Secretaria de Saúde, Secretaria de Desenvolvimento Social, Conselho Estadual da Criança e do Adolescente, representantes da sociedade civil, dentre outros. Dessa forma, considerando a existência dos órgãos supracitados, esta Parlamentar entende que não há razão para se criar mais um Comitê para exercer funções já prestadas por outras equipes, fato que implicaria um desperdício de recurso público e de força de trabalho de funcionários pagos pelo Poder Público. A criação de diversos conselhos e comitês, compostos pelos mesmos profissionais, acaba por gerar uma sobreposição de instâncias, o que prejudica até mesmo o exercício das funções originais de seus integrantes*” ^30^.

Merece destaque o argumento utilizado que ressalta a existência de um comitê intersetorial para a primeira infância. Esse argumento torna evidente o reconhecimento da intersetorialidade para formulação de políticas públicas, para um público e uma problemática diversa. Não parece plausível supor que um comitê intersetorial para a primeira infância possa atuar sobre homicídios de crianças e adolescentes, especialmente se considerarmos a centralidade das mortes decorrentes de intervenção policial e o perfil dos adolescentes vítimas de homicídio em São Paulo. Assim, esse veto, que reconhece a intersetorialidade como estratégia de gestão importante, recusa a intersetorialidade como resposta aos homicídios de crianças e adolescentes.

Para além de vetar o art. 7º, que previa a criação do comitê enquanto instância de coordenação, articulação e monitoramento da Política Paulista de Prevenção das Mortes de Crianças e Adolescentes, foram vetados outros importantes artigos, conforme demonstrado no Quadro 4.

**Quadro 4:** Vetos ao *Projeto de Lei nº 382/2022*. Estado de São Paulo, Brasil.

SEÇÃO	VETOS *	TEOR
IV - Das Diretrizes (art. 6º)	Inciso IV - vetado	“*Ampliar o investimento público em ações e programas que contribuam para a prevenção das mortes violentas de crianças e adolescentes*”
	Inciso VI - vetado	“*Estabelecer indicadores e metas específicas para o monitoramento das mortes violentas de crianças e adolescentes*”
V - Do Comitê da Política Paulista de Prevenção das Mortes Violentas de Crianças e Adolescentes (art. 7º e 8º)	Toda a seção	Tratava da criação do Comitê da Política Paulista de Prevenção das Mortes Violentas de Crianças e Adolescentes suas atribuições e representação
VI - Das Políticas de Prevenção à Morte Violenta e Resposta (art. 9º, 10º, 11º, 12º, 13º e 14º)	Art. 12º - vetado	“*Para as hipóteses que ainda não tenham sido avaliadas ou incluídas em programas específicos de prevenção à morte, poderá ser criado núcleo próprio de proteção provisória para atendimento e avaliação aos casos dessa natureza*”
	Parágrafo único - vetado	“*A proteção provisória de que trata o caput deste artigo visa o acolhimento emergencial, em caráter transitório, de crianças, adolescentes e familiares que aguardam sua inclusão nos programas de proteção, ou que se encontrem em situações que exigem a intervenção emergencial, com a finalidade de resguardar a incolumidade dos pretensos usuários, tendo em vista concreta situação de risco atual e iminente ofensa à sua vida ou integridade física*”
	Todo o art. 14º	Tratava das atribuições e responsabilidades das Secretarias e Instituições de Justiça
VII - Das Políticas de Segurança Pública e Interfaces com a Proteção à Vida de Crianças e Adolescentes (art. 15º, 16º e 17º)	Art. 17 - vetado	“*O Ministério Público, o Poder Judiciário e a Defensoria Pública, na elaboração de suas respectivas propostas orçamentárias, poderão prever recursos para a criação e manutenção da equipe de atendimento multidisciplinar voltadas ao atendimento de crianças, adolescentes e suas famílias, relativos a episódios de mortes violentas, nos termos da Lei de Diretrizes Orçamentárias*”
IX - Das Disposições Orçamentárias (art. 21º)	Todos os artigos da seção	“*O Estado informará à sociedade, anualmente, bem como consolidará na lei orçamentária, a soma dos recursos aplicados no conjunto de programas e serviços voltados à prevenção e redução da morte violenta contra crianças e adolescentes e o percentual estimado que os valores representam em relação ao respectivo orçamento realizado*”

Fonte: São Paulo ^24^.

* Seções I, II, III, VIII e X: sem vetos.

As questões que foram vetadas faziam menção a qualquer tipo de investimento com previsão orçamentária, ao estabelecimento de metas e indicadores para a avaliação da política, bem como a criação de mecanismos para tal. Sobre este último, esperava-se que a função fosse realizada pelo comitê, a partir das instituições que o integrariam (Quadro 2). As justificativas para esses vetos por parte do Governo do Estado foram: “*...tais preceitos exorbitam o exercício das competências parlamentares, não guardando a necessária concordância com as limitações decorrentes do princípio da separação de poderes (artigo 2º da Constituição Federal, e artigo 5º, ‘caput’, da Constituição Estadual). De fato, tais preceitos não se limitam a estabelecer princípios ou diretrizes, mas criam órgãos, atribuições e impõem ao Administrador ‘como fazer’, suprimindo do Governador a margem de apreciação que lhe cabe na concretização dos objetivos de que trata o projeto, de modo a contrariar as prerrogativas do Chefe do Poder* (...)*. Mostram-se também incompatíveis com a ordem constitucional os preceitos que pretendem disciplinar o conteúdo das propostas orçamentárias do Poder Executivo, do Poder Judiciário, da Defensoria Pública e do Ministério Público, uma vez que cuidam de matéria orçamentária, de iniciativa legislativa reservada ao Chefe do Poder Executivo*” ^31^.

Ainda que se considerem as justificativas, os vetos a esses artigos lançaram por terra o esforço de discussão e pactuação empreendido pelo CPPHA até então. No que diz respeito às responsabilidades institucionais na prevenção dos homicídios de jovens no estado, permaneceram apenas o inciso IV, do art. 5º, relativo ao fortalecimento do Programa de Proteção a Crianças e Adolescentes Ameaçados de Morte (PPCAAM); o parágrafo único do art. 16º, sobre as Guardas Civis Metropolitanas e sua atuação a partir do que lhes coubesse, e as atribuições da Secretaria de Segurança Pública e das Polícias Civil e Militar nos artigos 15º e 16º; e ao Ministério Público a prerrogativa de ser notificado em todo caso de morte violenta de crianças e adolescentes, art. 18º, conforme disposto no Quadro 3.

Em seu conjunto, os vetos ao PL nº 382 concorreram para o esvaziamento da - ainda que tímida - perspectiva intersetorial da Política Paulista de Prevenção das Mortes Violentas de Crianças e Adolescentes no que se refere à instituição de representação efetiva de atores públicos e da sociedade civil, ao compromisso de secretarias e instituições de justiça para com a agenda de prevenção e à distribuição de responsabilidades e atribuições. A lei acabou por instituir que a prevenção de homicídios de jovens no estado deve ficar à cargo da Secretaria de Segurança Pública e suas forças policiais, com alguma intervenção protetiva do PPCAAM, para os quais, entretanto, não se poderia garantir orçamento.

## Considerações finais

A análise da *Lei nº 17.652/2023*, que instituiu a Política Paulista de Prevenção das Mortes Violentas de Crianças e Adolescentes, indicou limites importantes ao exercício da intersetorialidade, à articulação interfederativa e à previsão orçamentária. O primeiro diz respeito ao fato de que as iniciativas se apresentaram mais como “a soma de todos os esforços”, ou antes, o agrupamento de ações e estratégias dos setores isolados que, eventualmente, em determinados temas - com destaque para a evasão escolar - poderiam se encontrar. Não foram estabelecidas maneiras de tornar a prevenção de homicídios de jovens um orientador transversal a todos os setores, buscando uma integração efetiva entre as ações que eles já desenvolvem, e não foram desenvolvidos mecanismos para a constituição de novas iniciativas intersetoriais.

Os vetos ao PL nº 382, no que diz respeito à constituição do CPPHA e, consequentemente, à participação de secretarias e instituições de justiça na Política Paulista de Prevenção das Mortes Violentas de Crianças e Adolescentes, refletem a dificuldade em reconhecer e atribuir responsabilidades a todos os setores, instituindo que esse é um problema que compete à aos setores tradicionalmente envolvidos com a questão, como segurança e justiça, uma vez que os outros atores foram vetados no texto.

A articulação interfederativa foi também um problema, dada a tímida presença de representação dos municípios, entes importantes para a gestão de políticas públicas nos territórios. A ausência das cidades acarreta inúmeras dificuldades para o estabelecimento de políticas, seja pela inadequação de determinadas propostas originadas no Governo Estadual, seja em impossibilidades de recursos financeiros.

A *Lei nº 17.652/2023* é exemplar também no que diz respeito ao veto dos artigos que instituíam a garantia de orçamento para a Política Paulista de Prevenção das Mortes Violentas de Crianças e Adolescentes. Se, de um lado, foram retirados todos os artigos que previam a participação de secretarias e instituições de justiça atuando na política, o que poderia ser compreendido como razão para a não necessidade de orçamento, por outro, o veto ao orçamento restringiu também a atuação das Polícias Civil e Militar, Guarda Civil Metropolitana e PPCAAM que, mesmo com atuações restritas e reduzidas a um ou dois parágrafos, continuaram na Lei, mas sem previsão de orçamento.

Para superar os desafios da construção de políticas efetivamente intersetoriais, necessárias à prevenção dos homicídios, nos parece necessária a criação, no âmbito do Executivo, de um gabinete de gestão intersetorial voltado à formulação e à implementação de políticas para a prevenção de homicídios e violência. Tal mecanismo deveria incluir representantes de todas as secretarias envolvidas, sem subordinação a nenhuma das pastas participantes para evitar os tensionamentos tradicionalmente existentes.
